# Book Reviews

**Published:** 1828-04

**Authors:** 


### CRITICAL ANALYSES.

Quæ laudanda forent, et quæ culpanda, vicissim

Illa, prius, cretâ; mox hæc, carbone, *notamus*.—PERSIUS.

*Pathological and Practical Researches on Diseases of the Brain and the Spinal Cord.* By JOHN ABERCROMBIE, M.D. Fellow of the Royal College of Physicians of Edinburgh, &c.—8vo. pp. 444. Waugh and Innes, Edinburgh, 1828.

(Concluded from p. 247.)

UNDER the head of *Inflammation of the Central Parts of the Brain*—the corpus callosum, septum lucidum, fornix, and the membrane lining the ventricles,—we meet with numerous cases illustrating the symptoms and pathology of the various forms of *acute hydrocephalus*. In common with the ablest pathologists of the present day, the author considers this disease as essentially inflammatory, the effusion being merely the result of the previous action, and not always present in cases which pass through all the usual symptoms of hydrocephalus. As there can now be little doubt that the leading principle to be entertained, in reference both to the pathology and treatment of this complaint, is its inflammatory nature, we think that the term hydrocephalus might be advantageously discarded from medical language, and one more appropriate adopted. In a medical point of view, it is manifestly unscientific, and its influence on practice more than doubtful. Moreover, we are sure that, were the term dropped entirely by medical men in common parlance, they would often be spared some mortification, and perhaps loss of reputation, by not treating a disease under the name of *water in the brain*, when, on inspection after death, not a drop of water can be found.

According to our author, the disease presents itself under two different forms. In the one, the inflammation appears to be seated in the membrane lining the ventricles; in the other, in the white matter forming the corpus callosum, septum lucidum, and fornix. In the former case, we have effusion into the ventricles, often with deposition of floculent matter on their inner surface, or surrounding the choroid plexus; or ramollissement of the adjoining cerebral substance. In the latter case, the affection presents itself in the ramollissement or white pulpy degeneration of the parts affected, the septum lucidum being perforated by an irregular ragged opening, owing to the softened portion having fallen out, and the fornix having lost its consistence and figure, or so much of its cohesion as to fall asunder at the slightest attempt to disturb it. Here the author reiterates his opinion that this peculiar degeneration is a product of inflammation, and refers to the facts presented by the cases detailed in the sequel. These distinctly show not only its frequent concurrence with effusion into the ventricles, but also its existence as the sole morbid appearance in decidedly marked cases of the disease. Some of the cases supply another satisfactory evidence of the inflammatory origin of this phenomenon, in the transition of the inflammatory state into that of ramollissement, distinctly visible in the same mass. This termination is, in our author's opinion, the result most to be dreaded; for, in regard to the mere effusion, were the parts otherwise healthy, he does not see any reason for considering it a hopeless affection, admitting the possibility of serous fluids being absorbed from the ventricles; a supposition which he thinks is warranted both by the analogy of other serous cavities, and by what we actually see take place in the brain itself. That absorption does take place in the brain, whatever the particular apparatus may be by which the function is carried on, he thinks is satisfactorily proved by the gradual disappearance of coagula from its substance and cavities. Unfortunately, however, the establishment of this fact would add little to our prospect of curing the disease, since effusion, uncombined with consequences more destructive and irremediable, is extremely rare; two cases only having occurred to our author in which he found serous effusion in the ventricles, without any other morbid alteration.

With all practical writers on the subject, we find the author alluding to the very proteo-form character of the disease, and the extreme difficulty of connecting the phenomena with the particular morbid condition of the parts affected.

"It is unnecessary to multiply cases which present no particular variety in the phenomena; those which have been described will probably seem sufficient to illustrate the principal forms of this affection, and at the same time to exemplify some of the most remarkable varieties in the symptoms. From a fair and candid review of the whole subject, I think we can have little hesitation in concluding, that this is the ordinary form of the disease, which is commonly called acute hydrocephalus; that it is originally an inflammatory affection, chiefly seated in the substance of the central parts of the brain; that it generally terminates by ramollissement of these parts, combined with serous effusion in the ventricles; and that it may be fatal by the ramollissement alone, even of small extent, but with all the symptoms which are commonly considered as characteristic of acute hydrocephalus. The cases likewise exemplify various important varieties in the symptoms. We have seen in some of them perfect coma of long continuance, without any effusion; and, in others, extensive effusion without any degree of coma. We have seen, again, the coma entirely removed, and yet the disease go on to its fatal termination. We have seen every variety in the state of the pulse, of the vision, and of the intellectual functions; and we have seen the disease run its course without any complaint of pain, or any symptom indicative of danger, until the patient was unexpectedly found in a state of profound coma. These, and many other varieties, presented by the cases which have been described, show us the danger of being guided by system in our diagnosis of affections of the brain, and the necessity that there still is for extensive and careful observation of facts in regard to this class of diseases." (P. 142.)

This acknowledgment is, we confess, humiliating, but it is honest, and far less likely to retard the attainment of truth than is the assumption of a certainty in diagnosis, to which our present stock of experience proves that we have no title.

The important position demonstrated by the cases detailed under this head, is the inflammatory nature of hydrocephalus; and the obvious conclusion to which they lead, is that our practice should be directed principally to subduing the inflammation at its earliest period, and preventing it from passing into effusion and the other results, particularly ramollissement, which we have seen to be a fatal termination, though of small extent, and without effusion.

A short section, which concludes the first part of the work, is allotted to the *treatment* indicated in this, as well as in the other forms of inflammatory affections of the brain. The remedies, according to our author, are few and simple, and their success will in a great measure depend on their being employed at an early period, and in the most decided manner. Those on which we must chiefly rely are blood-letting, general and topical, active purgatives, and cold applications to the head. We have too often to regret the inefficiency of these measures, even when judiciously employed; but we are not without a sufficient proportion of recoveries under the most unpromising circumstances, and the cases which thus terminate favourably hold out every encouragement to proceed with vigour in the treatment of a class of diseases, which, after a certain period of their progress, we are too apt to consider as hopeless. Next to the timely employment of the remedies, the ground of prognosis in particular cases will depend on the activity of the symptoms: the more these approach to the character of active inflammation, our prospect of cutting them short will be the greater; the more they partake of the low scrofulous inflammation, it will be the less.

Among the cases selected by the author to illustrate the treatment, we find examples of the disease under the most unpromising circumstances, some showing it arrested in the early or acute stage, and others at a more advanced period, after the supervention of coma and other symptoms which are usually considered as irremediable. In many cases general bleeding is employed, sometimes with immediate and permanent effect; in others a repetition of it is necessary to insure success. Active purging appears to be applicable to all cases, and, according to our author's experience, more recoveries from these head affections take place under strong purgation, than under any other mode of treatment. The effect of blistering in the early stages is rather doubtful: they ought at no period to be employed so as to interfere with a more decidedly useful topical remedy, the application of cold to the head. In acute cases, the most effectual mode of applying it is by a stream of cold water directed against the crown of the head, and continued until the effect is obtained. Applied in this way, it is an agent of such power that it requires to be used with much discretion. Under the operation of it, our author has seen a strong man thrown, in a very few minutes, into a state approaching to asphyxia, who immediately before had been in the highest state of maniacal excitement, with a morbid increase of strength defeating every attempt of four or five men to restrain him. Another case is given, showing the immediate effect of the remedy in a different modification of disease. A strong plethoric child, after one day of fever, restlessness, and oppression, fell rather suddenly into a state of perfect coma. When seen by our author, she was lying on her back, quite insensible, and with her face flushed and turgid. She was raised into the sitting posture, and a stream of cold water directed against the crown of the head, when, in a few minutes, or rather seconds, she was completely recovered, and next day was in her usual health. The same remedy he is in the habit of using, with the best effect, in the convulsive attacks of children, in preference to the warm bath; the indiscriminate employment of which he considers as being highly injurious.

The efficacy of mercury in hydrocephalus our author deems extremely questionable, and its employment during the first or more active stages decidedly injurious. We are inclined to dissent from this low estimate, or rather this sweeping condemnation, of an agent of such acknowledged power in arresting inflammatory action and its effects in other textures, membranous as well as parenchymatous. Many recoveries from head affections take place under its use, which we are warranted, by the analogy presented in croup, iritis, and hepatitis, to ascribe to its controlling influence.

*Tubercular Disease of the Brain.*—Our notice of the cases described under this section must be brief, and we can do no more than refer the reader to those relating to certain affections of the bones of the cranium and affections of the pericranium; all of which are extremely interesting.

Tubercles of the brain, where we have an opportunity of observing them in their early stage, from the patient having died of some other disease, present the same characters as in other parts of the body; and are, in fact, very often combined with tubercular diseases in other organs. The symptoms attending their early existence are often exceedingly obscure and variable; perhaps little more than a tendency to headache, which sometimes assumes the appearance of periodical, or what is called sick headache. The symptoms may go on for a long time in this state, until, either unexpectedly, or from some exciting cause, the affection takes on a more active character, and is speedily fatal, with symptoms of oppression; and exhibiting, on dissection, along with tubercular masses, the most complete evidence of general inflammation of the cerebral substance. These cases are generally very protracted: in some the symptoms are severe, particularly the headache, which is often excruciating and remarkable for its intermissions and the regularity of its periodical recurrence, the intervals varying from a day to a week or a longer term. The fatal termination is sometimes sudden and unexpected, while, on dissection, extensive disease is discovered. A most remarkable instance of this kind is given, in which the extent of destruction of brain, from suppuration and pulpy degeneration, could scarcely be supposed compatible with mere existence, although the patient spent the evening preceding her death cheerfully with a party at the house of a friend. The subject of this case had laboured for many years under symptoms denoting serious affection of the brain.

### *Of the Apoplectic Affections.*

In the investigation of apoplectic affections, we encounter the same difficulties which have hitherto rendered the pathology of inflammatory diseases of the brain so imperfect and unsatisfactory; viz. the want of uniformity in the appearances presented on dissection, and the absence of that correspondence between the symptoms and morbid actions so desirable with a view to judicious treatment in all diseases. A person previously in perfect health falls down suddenly, deprived of sense and motion, and dies after lying for some time in a state of coma. We find, on examination, a large coagulum of blood on the surface of the brain, or filling its ventricles, and the phenomena of the disease appear to be distinctly accounted for. Another person is cut off with the same symptoms, and we expect to find the same appearances; but nothing is met with except serous effusion, and that in small quantity. A third is seized in the same manner, and dies after lying for a certain time in a state of coma, from which nothing can rouse him for an instant, and, on the most careful examination, we cannot detect in the brain the smallest deviation from the healthy structure. Various speculations have, from time to time, been broached, to account for those cases in which no morbid lesion could be discovered on dissection, most of which are now deservedly forgotten, and that at present most generally entertained, viz. a temporary derangement of the circulating system of the brain, is beset with so many difficulties, that the author presents his own views on the subject, as "conjectures," in a separate section, not wishing to incorporate speculations, in a great measure hypothetical, with that part of the inquiry which embraces the history of the disease under its various aspects, the different morbid appearances disclosed by dissection, and the treatment.

The cases of apoplexy and paralysis detailed in the work amount to upwards of forty, and are arranged under three heads: 1, those which are immediately and primarily apoplectic; 2, those which begin with a sudden attack of headache, and pass gradually into apoplexy; 3, those which are distinguished by palsy and loss of speech, without coma. These three forms frequently pass into one another, but occur so often distinct as to warrant the arrangement thus proposed.

I. The first class includes those cases of sudden attack of apoplexy, in which the patient falls down as in a profound sleep, deprived of sense and motion, with slow pulse, stertorous breathing, and, generally, flushed face. From this state the patient may speedily and perfectly recover; or he may die after the lapse of a shorter or longer period. On dissection, we sometimes, though rarely, find extravasation of blood ; more generally serous effusion, though not in great quantity; but in many cases no morbid appearance whatever can be detected to account, satisfactorily, either for the attack or the fatal event.

Three or four cases are given from the author's experience, and several on record referred to, in illustration of that modification of apoplexy which is fatal without leaving any morbid appearance that can be discovered in the brain.

Under this head are also detailed those cases in which effusion of serum is the only or prominent appearance found after death, constituting what has usually been termed *serous apoplexy*, but which effusion the author is unwilling to regard as the cause of the apoplectic symptoms, but rather a result of some previous derangement of the circulation in the brain, to which also he thinks the cases first described ought to be referred. Dr. Abercrombie is also of opinion, with some authors, that the received division of apoplexy into *sanguineous* and *serous* is unsupported by observation, and conducive to bad practice; and certainly, whether we admit, or not, effusion to be a primary and sole cause of apoplexy, the cases described by the author, as well as by other writers, show that there is no ground for the distinction that has been drawn between the two forms; for the characters assigned to serous apoplexy are often found as the prevailing features of cases purely sanguineous, such as the advanced age of the patient, paleness of the countenance, and a feeble state of the circulation; while, on the other hand, many of the cases which terminate in serous effusion exhibit, in their early stages, all the symptoms usually assigned to sanguineous apoplexy.

II. The cases referred to the second class are not primarily apoplectic, or if, as in the first class, the patient falls down suddenly deprived of sense and motion, he speedily (in a few minutes or seconds, perhaps,) recovers, and is able to walk about. The prominent symptoms at the commencement are uneasiness or violent pain in the head, paleness of the face, weak pulse, generally sickness, and sometimes vomiting. The patient continues in this state for some time, oppressed but sensible, when, after the lapse of a few hours, the heat and the natural colour of the face return, and the pulse improves in strength; but the oppression gradually increases, the speech becomes slow and embarrassed, and at last fatal coma is established. The time from the first attack to the establishment of coma, or the complete apoplectic condition, varies, in different cases, from a few hours to two or three days; there being always, however, a certain interval of sense, generally with violent complaint of pain, which distinguishes these cases from what may be strictly called the apoplectic attack, which is characterised by immediate and complete loss of sensibility. Cases of this class generally terminate fatally, and, according to our author, there is less ambiguity in the morbid lesions than in the cases of simple apoplexy; extravasation of blood, to some extent, being generally found on dissection. These cases may therefore, he thinks, be safely attributed to the immediate rupture of a blood-vessel, without any previous derangement of the circulation; the rupture probably arising from preexisting disease of the artery at the place which gives way. It is supposed that, at the moment of the rupture, a temporary derangement of the cerebral functions takes place, which is soon, in a great measure, recovered from, but, the circulation going on, blood is at length extravasated in sufficient quantity to induce coma; the progress to coma depending, probably, on the size of the ruptured vessel and the remedial measures employed. We present the two following cases as examples of this modification of apoplexy. In the one, the coma supervened rapidly on the first attack; in the other, there was an interval of three days between the attack and the occurrence of coma; in both it will be remarked that the early symptoms were such as have been supposed to characterise serous apoplexy.

"A clergyman, aged fifty-five, while delivering his sermon during the morning service of Sunday, 13th May, 1827, was observed to stop and put his hand to his head; he then attempted to go on, but talked indistinctly, and had evidently lost his recollection; he supported himself by grasping the side of the pulpit. Assistance being immediately given him, he was taken out, and at this time was speechless and paralytic of the right side, but appeared to be sensible. He became rapidly more and more oppressed, and, in about twenty minutes from the commencement of the attack, had become entirely comatose. From the time when he was taken down from the pulpit, he was pale and cold, and his pulse extremely feeble; and this state continued when I saw him, about an hour after the attack; so that, though a vein was opened, very little blood could be obtained. Gradually the circulation rallied, and in another hour a full bleeding was obtained, without relief. All the other usual remedies were employed without benefit. From the time when the coma took place, there never was the slightest abatement of it; he lay with his eyes shut, his countenance pale and sallow, but placid and without distortion; his pulse weak, the power of swallowing lost, the breathing at first stertorous, afterwards slow and oppressed. He lived in this state till Monday at mid-day, about twenty-four hours after the attack.

"*Inspection.*—There was extensive extravasation of blood in the left ventricle, which had passed partly into the right by laceration of the septum. It seemed to have made its way into the ventricle from the substance of the brain, on the outer and anterior part, where there was a large irregular lacerated cavity, full of coagulated blood, and communicating with the ventricle. All the arteries of the brain were extensively ossified." (P. 224.)

"A gentleman, aged eighteen, previously in good health, after using rather violent exercise in the forenoon, had returned home before dinner, and was sitting near the fire, when, without any warning, he started up, pushed his chair backwards with violence, exclaimed, 'Oh my head!' and instantly fell on the floor insensible, and slightly convulsed. I saw him within ten or fifteen minutes after the attack. By that time he had recovered his recollection, was sitting on a chair, and was quite distinct. His face was extremely pale, and his whole body cold and shivering; he complained of severe headache, and his pulse was weak and rather frequent. Bloodletting was immediately employed, and his pulse improved under it. It was repeated after a few hours, with the addition of purgatives and the other usual remedies. The coldness and paleness went off after some time, and he then complained only of severe headache, with a feeling of stiffness of his neck, and pain extending downwards along the cervical vertebræ; his pulse was rather frequent and of good strength. He continued in this state for two days, the headache varying very much in degree, and frequently complained, chiefly of his neck; his pulse was frequent, 120 or more, and of good strength; the other functions were natural; he was quite distinct; had the use of all his limbs, and could get out of bed with little assistance, and sit up a considerable time. On the third day he began to be more oppressed, and a little confused and forgetful; the other symptoms as before. On the fourth he sunk very gradually into coma, and died on the 5th. His pulse had continued from 120 to 140; there had been no paralytic symptom; but on the fifth day there was repeated convulsion. Bloodletting, and all the other usual remedies, had been employed without benefit.

"*Inspection.*—All the ventricles of the brain were completely filled with coagulated blood. In the substance of the left hemisphere there was a cavity formed by laceration of the cerebral substance, filled also by the coagulum, and communicating with the ventricle. There was no other morbid appearance." (P. 229.)

There is reason to believe that, after the rupture has taken place, the hemorrhage may be arrested by the formation of coagulum, and, after some time, burst out afresh, and prove fatal. In such cases, the two extravasations can sometimes, on inspection, be distinguished from each other by their appearance.

The seat of the extravasation varies in different cases. The most common situation in which we meet with it is the substance of the hemispheres; in some cases it is confined to the ventricles only; and in others it is extensively spread over the surface of the brain, between the dura mater and arachnoid.

The most common source of the hemorrhage appears to be the rupture of a vessel of moderate size in the substance of the brain, from which the blood bursts its way by laceration either into the ventricles or to the surface, though in general it is not possible to trace the hemorrhage to its very origin. Disease of the vessels is supposed to precede and lead to this rupture, and very generally such previous disease will be found, on dissection, to exist: as ossification of the arteries in various places, or the peculiar earthy brittleness which Scarpa describes as leading to aneurism. In some cases, again, the inner coat of the arteries is much thickened, of a soft pulpy consistence, and easily separated. Ossification in the course of the arteries of the brain is by no means uncommon in old people, but the same condition may occur even before the middle term of life, and a case is given which shows that extensive ossification of the arterial system of the brain, with thickening and partial separation of the inner coat of the vessels, may exist for several years before it leads to extravasation and fatal apoplexy, though the patient, as might have been expected, laboured under considerable derangement of the cerebral functions. The hemorrhage sometimes takes place from the superficial vessels, constituting the *meningeal apoplexy* of Serres, or from the vessels of the choroid plexus, in which case the coagulum will be found in the ventricles. The extravasation has also been traced to ulceration and rupture of one of the principal arterial trunks, as the b[illegible]c[illegible]lar artery; to rupture of one of the sinuses; and to the bursting of small aneurisms of the cerebral vessels.

III. The third class of apoplectic affections which become the subject of our author's researches, comprehend the *Paralytic Cases.* The leading features of this class are hemiplegia and loss of speech, without coma. Some of those cases, indeed, which begin as a paralytic attack, pass, after some time, into apoplexy, and properly belong to the second class. The cases described in the present section are, on the contrary, primarily and in their after progress, paralytic. The attack, under proper treatment, may, like simple apoplexy, pass off speedily and entirely, or, like it, may prove fatal, and, on dissection, present no satisfactory appearance, or only serous effusion, often in small quantity. In some cases the recovery is gradual, the use of the limbs being restored after several weeks or months. In other cases, again, the palsy may be permanent, the patient being not altogether helpless, but living for many years with imperfect use of his speech and limbs; or, lastly, the patient makes no recovery at all, but remains confined to bed, and reduced to that grievous state of existence which can hardly be deemed a boon.

The morbid conditions of the brain connected with these varieties are considerably diversified. Some bear a close analogy to what the author styles *simple apoplexy*, in which a formidable attack takes place, and either speedily and entirely passes off, or proves fatal without leaving any traces, on dissection, of serious injury of the brain. Many cases can, after death, be distinctly traced to extravasation of blood of small extent, contained in defined cysts in the substance of the brain or under the membranes. Other cases, again, depend on ramollissement of the cerebral substance, or supervene on inflammation and its consequences.

We are presented, in the course of the inquiry, with a number of cases occurring in connexion with these several morbid conditions of the brain, but the uniformity of symptoms is not such as can enable us, in all cases, to discriminate them, during life, from each other.

Extravasation of blood appears to be a common cause of paralysis, and it will naturally be inquired why extravasation should in one case produce perfect apoplexy, and in another palsy. According to our author, it would appear that the difference of result, in this respect, depended on the extent of the extravasation: extravasation of a limited extent, so as to be confined to a cavity of moderate size, inducing palsy; but, when the cavity is greater, to the loss of sense and motion we have coma superadded, constituting the apoplectic state. Attempts, indeed, have been made to establish a connexion between the functions impaired or suspended and the particular portions of the brain which are affected. Serres, in particular, has endeavoured to trace this connexion, but subsequent authors have adduced cases to show that the concurrence of morbid lesions of certain parts with particular symptoms is by no means uniform or general. This appears to be the opinion of the author. The subject is, however, one of much interest, and deserving of farther prosecution, in reference both to our diagnosis of apoplectic affections and to certain parts of the doctrine of phrenology, which doctrine will probably stand or fall through the labours of pathologists.

The danger or irremediableness of the case does not appear to depend so much on the amount of the extravasation as on the condition of the surrounding cerebral substance. When extravasation of small extent proves fatal, it is in general connected with ramollissement of a portion of the cerebral substance around the cavity which contains the coagulum. On the other hand, when the cerebral substance surrounding the extravasation is in a healthy state, we find that coagula of a very great size are gradually and completely absorbed. The author gives several interesting cases illustrative of this curious fact. The absorption appears to commence at a very early period, but to advance very slowly. So early as thirteen or fourteen days from the attack, a change from the appearance of recent blood has been observed to take place, the coagulum being firm in its texture and of a dark brownish colour. In its farther progress, the coagulum assumes a fibrous texture, and the last portion that remains is a small mass of fibrine, of a slight reddish colour, which, after a certain time, also entirely disappears, leaving an empty cavity. When these changes are going on in the coagulum, the cavity in which it is contained becomes lined with a firm membrane; and, when the absorption is completed, we find this membrane forming a distinctly organised cyst, with numerous blood-vessels ramifying upon it. This cyst is generally empty, and, according to the French pathologists, it is sometimes obliterated.

No definite time can be assigned for the disappearance of the coagulum and the deposition of this new membrane. As the absorption goes on, the symptoms, in some cases, subside gradually, and entirely disappear; but more generally the improvement is but partial, and, even after the entire absorption, the patient may continue paralytic to the end of his life, the only morbid appearance presented on dissection being the empty cyst.

Of paralysis from ramollissement two cases only are given; this form of the disease not having fallen much under our author's observation, though it appears to be a frequent occurrence in the French hospitals. It is generally met with in the old and infirm, and we have already alluded to our author's view of this affection, viz. its identity with gangrene in other parts of the body, from failure of the circulation in consequence of disease of the arteries. The symptoms do not seem to differ remarkably from those which occur in the other cases of palsy; though, in general, it may be observed that they advance more slowly: one organ, as the tongue, being first affected, and then one or more limbs in succession. Pain in the affected limbs is also a frequent occurrence, and rigid contraction of them has been much insisted on as characteristic of the ramollissement. The patient generally dies comatose, or exhausted with symptoms of low fever.

Besides the above, we meet with other varieties of paralysis exhibiting peculiarities of symptoms, not easily [illegible]pplicable on any principles with which we are at present acquainted. In some cases there is loss of motion without loss of feeling; in some there is loss of motion on one side, and loss of feeling, without any diminution of motion, on the other. In other cases, again, the sensibility is morbidly acute, with intense pain of the affected limbs. Occasionally there is a depraved condition of sensation, cold bodies imparting the impression of heat, and vicè versâ. It does not appear that paralytic limbs are actually colder than healthy limbs, but they seem to lose, in some degree, that remarkable power possessed by living bodies in a healthy state, of preserving a medium temperature, so that they become hotter or colder than sound parts which have been exposed to the same temperature. The recent discoveries of Mr. Charles Bell are referred to as likely to throw light on this curious and obscure subject. The morbid condition of the mental faculties, frequently observed in connexion with paralysis, are not less curious and difficult of explanation. One of the most common is a loss of the memory of words; sometimes only of words of a particular class, as nouns, verbs, or adjectives; or the substitution of one word, or one name of an object, in the place of another; and a very singular circumstance in some cases of this kind is, that the patient always applies these words in the same manner. In other cases the patient invents words and names, which to strangers are unintelligible, but which he always uses in the same sense.

We have already incidentally alluded to the views entertained by the author relative to the state of the circulation in the brain in those cases of simple apoplexy in which the patient either speedily and entirely recovers from the most formidable symptoms, or dies without our being able to detect the smallest deviation from healthy structure. A temporary compression of the brain from undue accumulation of blood in the cerebral vessels has in these cases been usually deemed the efficient cause of the symptoms. To this it is objected that, on well-known philosophical principles, such a cause as accumulation cannot operate within the cavity of the cranium, which is a complete sphere of bone, exactly filled by its contents, by which construction the brain is shut up from atmospheric pressure, and from all influence from without except what is communicated through the blood-vessels which enter it. In an organ so situated it is, therefore, contended that the quantity of blood circulating in its vessels cannot be materially increased, unless something give way to make room for the additional quantity, because the cavity is already full; nor materially diminished, except something entered to supply the place which would become vacant. That this principle, so true in physics, holds good in the case of the cranial cavity, appears to be proved by several facts, particularly by the appearance of the brain in animals which have been bled to death. While, in these cases, all the other organs of the body have been found completely drained of blood and blanched, the [illegible] has presented, in this respect, its usual appearance, and in some cases even a state of the superficial veins approaching to congestion.

Dr. Kellie, whose experiments are referred to as the most satisfactory on this point, to the above facts adds the result of observations he made on two subjects who had been hanged: on dividing the scalp in these cases, the blood flowed in such quantities as to afford ample proof of congestion in the vessels exterior to the cranium, but nothing unusual was observed in the brain, except, indeed, that the pia mater was, upon the whole, paler and less vascular than it is commonly met with.

From these facts, the author thinks it highly probable that, in the ordinary state of the parts, no material change can take place in the absolute quantity of blood in the brain, and, as the quantity circulating in its vessels must be divided in a certain ratio between the arteries and veins, it is also probable that the healthy state of the brain will depend on the nice adjustment of the circulation in these two systems. Were accumulation to take place, from any cause, in one of the systems, the necessary consequence would be a corresponding diminution in the other. The arteries going to the brain, for example, might, under the influence of general plethora, be able to introduce more than the average quantity of blood into the head; but this would necessarily lead to a corresponding diminution of the quantity in the veins; and, again, if there be any considerable interruption to the return of the blood from the veins of the brain, such as might be caused by tumors on the neck, or certain diseases of the heart and lungs, a derangement would take place analogous to that supposed in the preceding case—an accumulation in the veins, with a corresponding diminution in the arteries. These views, it must be observed, are advanced by the author as "conjectures," and not pressed as incontestible explanations of the phenomena of apoplexy, though he is evidently disposed to adopt them in preference to the notion of pressure of the brain, which has hitherto been generally considered the efficient cause of apoplexy; but we confess we cannot easily separate the idea of pressure from the modes in which this derangement is supposed to take place.

There are some facts brought forward in this place which, apart from their application to any particular theory, possess considerable importance in a practical point of view: we allude to symptoms indicating deranged circulation within the head, such as headache, sense of fulness and confusion, throbbing, giddiness, and even coma, occurring in a state of the body the reverse of what is usually [illegible]ered apoplectic, as in an exhausted state consequent on hemorrhage, or excessive depletion, and in the last stage of debilitating diseases; conditions in which the general volume of blood is evidently much diminished. With reference to this subject, the author makes the following practical remarks; and we cannot avoid expressing our belief that, in theory as well as practice, we too often disregard the *apoplexia ab exinanitione* of the old writers, many distressing and alarming symptoms appearing not unfrequently to depend mainly, in the first instance, on a state of general debility, and necessarily requiring a modification of treatment adapted to this state of exhaustion.

"I have been repeatedly consulted under the following circumstances. A gentleman, accustomed to very full living, is seized with an apoplectic attack, or with symptoms indicating the most urgent danger of apoplexy; he is saved by bleeding and other free evacuations, and is kept for some time upon a very spare diet. His complaints are relieved, and, as long as he keeps quietly at home, he goes on without any uneasy feeling. But, when he begins to go abroad, he becomes liable to attacks of giddiness and confusion, generally accompanied by palpitation of the heart and an uneasy feeling about the præcordia. His pulse is now soft and rather weak, and his general appearance indicates the very reverse of plethora, and these symptoms are removed by a cautious improvement of his regimen. This curious fact I have repeatedly had occasion to attend to in the treatment of cases of this kind, and it has always appeared to me to be one of very great interest in reference to the pathology of the brain." (P. 310.)

*Treatment of Apoplexy.*—The remedies chiefly relied on by the author are bloodletting, general and topical; active purgatives; cold applications to the head; and the strict observance of the antiphlogistic regimen. Bloodletting, with a view to take off the impulse from the arteries of the brain, is recommended to such an extent as shall powerfully and decidedly affect the system, and its employment insisted on without any regard to the hypothetical division of apoplexy into sanguineous and serous; and the only cases illustrative of the treatment which he thinks it necessary to detail are such as occurred in old and infirm people, which might have been considered either as examples of serous apoplexy, or modifications of the disease not admitting of active treatment, yet under such treatment terminating favorably.

The same practice is said to apply equally to the early stages of paralysis. No general plan of treatment can be laid down for this affection in its more advanced stages. The restoration of paralytic limbs is in many cases the work of nature, probably consisting in the gradual absorption of the coagulum. In other cases, however, the recovery is so remarkably sudden as to lead to a suspicion that paralysis may depend on a cause less permanent and irremediable than any of those with which we are at present acquainted.

The last class of affections of the brain which come under review comprehends the *Organic Diseases,* which consist either in permanent changes of the brain itself, or new formations within the head. Under this class the author treats of tumors,—deposition of albuminous matter,—tubercular disease,—induration of the cerebral substance,—ossifications,—and hydatids. The symptoms attending these various morbid changes are not sufficiently uniform in their character to determine accurately the particular seat or nature of the affection. The author, however, gives a general outline of the most common modifications under which they present themselves, and several cases from his own observation, and from other sources, descriptive of each variety of disease.

Want of space prevents us from entering more fully into the subject of organic diseases, which is one of great importance from the protracted nature of the affections to which they give rise, as well as the extreme sufferings of the patient under them. Here, as elsewhere, the author presents a great mass of information, which will be advantageously consulted, [redacted] with a view to diagnosis, by those who may have to treat this obscure and too often unmanageable class of cases.

Want of space also prevents us from following our author through the remaining part of the volume, which treats very ably and fully of various diseases, acute and chronic, to which the spinal marrow and its envelopes are liable; but we would strongly recommend it to the attention of our readers. This portion of the inquiry evinces the same industry in collecting materials, and the same caution in making deductions, so conspicuous in the preceding investigations. Indeed, the whole work is rich in facts, and entitles the author to a high rank among the pathologists of the present day, who labour to enlarge our acquaintance with the phenomena of disease, and fix the principles and extend the usefulness of the healing art.

---

*Diseases of the Lungs; being the second Part of a Treatise on the Diseases of the Chest and on Mediate Auscultation.* By R. T. H. LAENNEC, M.D. Regius Professor of Medicine in the College of France, &c. (Continued.)

WE resume our analysis of the valuable work of LAENNEC with the Diseases of the Lungs, having in a preceding Number entered fully into those of the Bronchia.

Our author premises a short account of the observations of various writers respecting the intimate structure of these organs, and he is induced to regard the opinions of REISSEISSEN as most correct, who, by means of many microscopical examinations and mercurial injections, was led to believe that the bronchia are subdivided into a multitude of small canals, at their extremities terminating in culs-de-sac of a globular form, arranged somewhat like the component parts of a cauliflower. But, whatever be the intimate structure of the pulmonary tissue, if we examine the surface of a sound lung in a bright light, we can ascertain that its parenchyma, by the grouping together of a number of small vesicles, which are full of air, and separated from each other by white partitions. The largest of these vesicles do not exceed in size the third or fourth part of a millet-seed, and the partitions which divide these groups from each other cross under various angles, so as to form figures of very different shapes. It is along these lines that the black pulmonary matter is deposited, which is so frequently confounded with melanosis.

*Hypertrophy of the lungs* is the first specific disease mentioned by Laennec, and he begins with it as being the most simple morbid alteration to which our organs are subject. It is marked by increase in the size, and sometimes in the consistence of the part. It is not attended with any inconvenience, unless it happens to affect a part whose increased energy disturbs the functions of the body. In double organs, when one is destroyed, the other acquires increased activity, which after a time gives rise to augmentation of volume. With regard to the lungs, it was observed by Morgagni, that, when one lung was compressed, the viscus on the opposite side was sometimes increased in size; and, in fact, this occurs in every instance where one of the lungs is rendered useless for a certain time. When this takes place, the lung increases in volume, becoming at the same time firmer, more elastic, and more compact, so that, when the chest is opened, it will sometimes protrude, instead of collapsing.

*Atrophy of the lungs* is a state of coma, just the reverse of the preceding. They do not participate in general emaciation of the body, but diminish in size from the effects of pressure, whether applied externally or resulting from the growth of accidental productions internally.

*Emphysema of the lungs* is divided into the vesicular or pulmonary proper, and the interlobulary. The former of these is, next to hypertrophy, the simplest of all the organic lesions of the lungs, and consists merely in a dilatation of the air-cells. It long remained undiscovered, and Laennec himself for a long time looked upon it as extremely uncommon. He considered it more lately, however, as a frequent cause of asthma. Its anatomical characters may be described as an exaggeration of the natural condition, in which the size of the vesicles is increased till they become as large as millet-seeds, while some attain the magnitude of cherry-stones. These very large vesicles are probably produced, for the most part, by the partitions between several cells breaking down; sometimes, however, according to Laennec, they depend upon the dilatation of one individual vesicle. We remember ourselves to have seen them as large as hazel-nuts. Sometimes the air becomes extravasated under the pleura, forming large irregular vesicles on the surface, as large as a walnut, or even as an egg. To enable us to acquire a correct idea of this disease, the lungs must be inflated and dried: if they are then cut into slices, we perceive that the air-cells are more or less dilated, and some of them ruptured. When we blow into an emphysematous lung, the dilated cells seem to become flatter the more they are distended; a circumstance which is owing to the greater relative expansibility of the healthy cells, which rise to the level of the others. Laennec states his belief that, if we carefully examine the lungs of those who have long suffered from difficulty of breathing, from whatever cause, we shall almost always find some of the air-cells dilated. When the disease exists in a high degree, the lungs rise beyond the borders of the thorax, when this is opened, and the crepitation they afford is of a different character, owing to the air passing from the cells much more slowly than in the healthy state. When a single lung is affected, it becomes much more voluminous, so as occasionally to encroach upon the opposite side. Pulmonary emphysema is apt to supervene on very severe dry catarrh; and another occasional cause may be found in very great exertion of the lungs, as in blowing a wind instrument; perhaps, also, tumors developed in the chest, so as to press upon the bronchial tubes, may sometimes produce this affection.

The symptoms of emphysema of the lungs are rather equivocal. Dyspnœa is the principal, and is usually called asthma. The difficulty of breathing is constant, but aggravated by irregular paroxysms, and there is habitual cough. The disease frequently begins in childhood, and may continue many years; for it does not always prevent the patient from obtaining advanced age. The respiratory sound is inaudible over the greater part of the chest, while a very clear sound is produced by percussion. When existing in a high degree, according to Laennec, this disease may be recognised by a sign which he looks upon as pathognomonic, and which he describes under the name of the *dry crepitous rattle with large bubbles;* a sound which conveys the idea of air entering and distending lungs which had been dried: it resembles, in fact, the sound produced by blowing into a dried bladder.

Emphysema of the lungs being almost always the consequence of dry catarrh, is to be treated in a similar manner.

The *interlobular emphysema* is that which follows wounds, by which air becomes infiltrated in the cellular partitions which divide the air cells from each other. To have a correct idea of its anatomical characters, we must inflate the lung, pass a ligature above the affected part, and then dry it in the open air. If the preparation be then cut into slices, we observe the interlobular cellular substance, presenting the appearance of a series of cells, of unequal shape and size, all communicating with each other. The most common cause is the prolonged and forcible retention of the breath during powerful exertion. When the extravasation of air is confined to the lungs, it appears to be reabsorbed, and Laennec never saw a fatal result from this disease alone. He gives two cases in illustration.

"I. A young woman, convalescent from a severe acute catarrh, in the winter of 1823-4 presented the most evident signs of this disease on the right side, over a space larger than the hand. The dry crepitous rattle with large bubbles, the friction of ascent during inspiration and of descent during expiration, were heard strongly and distinctly. A sense of crepitation was also felt by the hand during the stronger inspirations: sometimes, however, this was altogether wanting, and at other times it could be excited by pressure in the intercostal spaces. The dyspnœa, which was considerable when I first saw the patient, gradually diminished, and the phenomena above mentioned gradually decreased with it. At the end of two months the young woman left the hospital. I saw her in the ensuing spring, and found her quite well, and without any symptom of emphysema.

"II. A man, aged twenty, came into the clinical wards the 9th May, 1825, affected with a very severe catarrh of three weeks' duration. At this time he had all the symptoms of the acute suffocative catarrh,—extreme dyspnœa, tracheal rattle, high fever. The chest, on percussion, sounded pretty well throughout, but perhaps somewhat imperfectly on the left back; the sound of respiration was feeble or moderate every where; mucous, sonorous, and sibilous rattles, singly or conjoined, existed in different points, and a slight crepitous rattle, with bronchial respiration, was perceived at the roots of the left lung.

"The mucous rattle was felt by the hand in several places, particularly on the sides. There was also a sub-crepitous rattle at the root of the right lung. These latter signs being indicative of an incipient double peripneumony, although there was as yet hardly any expectoration, I ordered eight ounces of blood to be taken from the arm, and prescribed emetic tartar in large doses. This was borne moderately well, and produced considerable action on the bowels, without vomiting.

"On the 12th, the expectoration was glutinous and tinged with blood, and there was severe pain of the right side, in consequence of which cupping glasses were applied. But the tracheal rattle and dyspnœa were somewhat diminished, and it was further discovered by the stethoscope that the peripneumony had not advanced in those points where the crepitous rattle had been found, while it had retrograded in those where the bronchial respiration had been perceptible; this mark of hepatization being now superseded by a crepitous rattle.

"On the 14th, the tracheal rattle being less, and the strength improved, I added some syrup of poppy to the antimonial mixture, and allowed the patient more soup. After this time, the fever, diarrhœa, and dyspnœa did not return; the respiration began to be pretty distinct over the whole chest, and, although it was accompanied by different kinds of rattle, the crepitous was not among them.

"On the 17th, the patient was quite convalescent.

"On the 20th, on examining the chest more closely, I discovered the friction of ascent and descent on both sides of the chest, and also the dry crepitous rattle with large bubbles; the former most marked on the left, the latter on the right side. I made, in consequence, the following addition to the diagnostic ticket:—

"Emphysema interlobulare partium inferiorum utriusque pulmonis."

"This patient left the hospital on the 29th, the signs of the emphysema, although daily decreasing, being still very well marked. At the end of three weeks, he called upon me, when I found him quite well, and without any of the stethoscopic signs above mentioned." (P. 173.)

*Œdema of the lungs* is constituted by an infiltration of serum into the substance of the organ, so as to diminish its permeability to the air. It is seldom a primary disease, but comes on with other dropsical affections: the most severe cases that Laennec ever met with occurred during a temporary convalescence from peripneumony. Although it is commonly merely a consequence of other diseases, yet in other instances it seems to be idiopathic; and it is conjectured by our author to be a frequent cause of death in children after measles. When it occupies the whole of one lung, this becomes of a pale grey colour, is more dense than natural, and does not collapse on opening the chest. It is, however, nearly as crepitous as before. The symptoms of this disease are not well marked. Impeded respiration, with slight cough and watery expectoration, are generally present. Percussion scarcely affords any assistance, but the stethoscope furnishes two diagnostic marks.

"The respiration is much feebler than might be expected, from the great dilatation of the thorax; and there is at the same time a slight crepitation, as in the first degree of peripneumony, more like a rattle than the natural sound of respiration. This crepitous, or rather subcrepitous rattle, is more humid than in peripneumony, and the bubbles appear larger. It must be admitted, however, that it is sometimes difficult to distinguish these two diseases by the stethoscope alone, without taking into account the general symptoms. When the œdema is very general and in a high degree, the natural sonorousness of the chest is very perceptibly lessened; and in these cases there is slight bronchophonism, particularly at the roots of the lungs. But we can almost always distinguish the œdema from the incipient peripneumony, by the long continuance of the crepitous rattle, and the absence of the general symptoms of inflammation in the former disease." (P. 176.)

*Pulmonary Apoplexy.*—The principal symptom of this disease is hemorrhage from the lungs, generally of a severe kind, and entirely distinct from those milder cases of hæmoptysis, of which we spoke in the preceding Number.

"Those cases, however, of violent and extreme hemorrhage, which often resist all medical treatment, arise from a very different and more dangerous cause. In these, some part of the pulmonary substance has undergone great changes, being indurated to a degree equal to the completest hepatization. The induration, however, is very different from the inflammatory affection of the lungs distinguished by this term. It is always partial, and scarcely ever occupies a considerable portion of the lungs, its more ordinary extent being from one to four cubic inches. It is almost always very exactly circumscribed, the induration being as considerable at the very point of termination as in the centre. The pulmonary tissue around is quite sound and crepitous, and has no appearance whatever of that progressive induration found in the peripneumonic affection. The substance of the lung is, indeed, often very pale around the hæmoptysical induration; sometimes, however, it is rose-coloured, or even red, as if tinged with fresh blood; but, even in this case, the circumscription of the indurated part is equally distinct. The indurated portion is of a very dark red, exactly like that of a clot of venous blood. When cut into, the surface of the incisions is granulated as in a hepatised lung; but, in their other characters, these two kinds of pulmonic induration are entirely different. In the second degree of hepatization, along with the red colour of the inflamed pulmonary tissue, we can perceive distinctly the dark pulmonary spots, the blood-vessels, and the fine cellular intersections; all of which together give to this morbid state the aspect of certain kinds of granite, as has been already observed. In the induration of hæmoptysis, on the contrary, the diseased part appears quite homogeneous, being altogether black, or of a very deep brown, and disclosing nothing of the natural texture of the part, except the bronchial tubes and the larger blood-vessels. The latter have even lost their natural colour, and are stained with blood. The veins of the affected part, and also those adjoining, are sometimes filled with a firmly coagulated and half-dry blood; a kind of infarctus, which will be noticed afterwards when we come to treat of the diseases of the pulmonary vessels. In scraping the incised surfaces of these parts, we can detach a small portion of very dark half-congealed blood, but in a much less proportion than we can press out the bloody serum from a hepatised lung. The granulations on the incised surfaces have also appeared to me larger than in cases of hepatization. Sometimes the centre of these indurated masses is soft, and filled with a clot of pure blood." (P. 184.)

The principal symptoms are oppression, cough, and pain of the chest, with expectoration of blood, which may be either bright and frothy or black and coagulated. It is generally very copious, returning by fits, with cough, oppression, and coldness of the extremities; occasionally there is vomiting. The quantity of blood expectorated is sometimes very great: Laennec has known ten pounds lost in forty-eight hours.

The treatment consists in free venesection at the onset of the disease, and our author also recommends purgatives; he has also tried tartar emetic in one or two desperate cases, and has never known any bad effect result from it. "In the present case, still more than in the bronchial hemorrhage, we must not give astringents and bitters until the disease has assumed a chronic character."

---

*A Grammatical Introduction to the London Pharmacopœia and Preface; or, the Student's Self-Instructor. Containing a Latin Grammar, Exercises, and Vocabulary; a Syntactical Analysis of the Pharmacopœia and of the Preface; and an Appendix of Words most frequently occurring in Physicians' Prescriptions.* By S. F. LEACH. Second Edition, considerably enlarged.—18mo. pp. 180. Anderson, London, 1828.

WE are glad to find that Mr. LEACH has been encouraged to publish a second edition of the above little volume. Nothing was ever more called for than a work of this description, which, whilst it teaches the grammatical construction of the Latin language accurately and fully, does so by taking all its examples from the Pharmacopœia, as well as the declension of its nouns, adjectives, &c.; so that the pupil learns his text-book at the same time that he imbibes a knowledge of the language in which he is obliged to read it when passing his examination. The second edition differs considerably from the former, containing an entirely new division, entitled "Directions for Parsing, and a Syntactical Analysis of the Pharmacopœia and Preface:" this is so complete that the student, who shall thoroughly understand this book, cannot fail to possess a very fair and accurate grammatical knowledge of the structure of the Latin tongue. We beg, therefore, strongly to recommend it to those young men who have not been fortunate enough to receive a classical education. It is a short, but fair and legitimate, road to attain an indispensable point: it will not only certainly effect this, but it may even excite a thirst for classical knowledge, and lead the pupil to quench that thirst from the pure fountains of the ancient writers.

