# Errata

**Published:** 1828-05

**Authors:** 


### ERRATA.

P. 339, 8th line from the bottom, *for* bacelar *read* bisilar.
340, 23d ditto — *for* substances *read* substance.
342, 12th ditto from the top, *for* applicable *read* explicable.
344, 19th ditto — *for* rendered *read* considered.
345, 4th line from the bottom, *for* both with a view to diagnosis, *read* with a view both to diagnosis and practice.

